# Objectively measured physical activity and sedentary time in youth: the International children’s accelerometry database (ICAD)

**DOI:** 10.1186/s12966-015-0274-5

**Published:** 2015-09-17

**Authors:** Ashley R. Cooper, Anna Goodman, Angie S. Page, Lauren B. Sherar, Dale W. Esliger, Esther MF van Sluijs, Lars Bo Andersen, Sigmund Anderssen, Greet Cardon, Rachel Davey, Karsten Froberg, Pedro Hallal, Kathleen F. Janz, Katarzyna Kordas, Susi Kreimler, Russ R. Pate, Jardena J. Puder, John J. Reilly, Jo Salmon, Luis B. Sardinha, Anna Timperio, Ulf Ekelund

**Affiliations:** Centre for Exercise, Nutrition and health Sciences, School for Policy Studies, University of Bristol, Bristol, UK; National Institute for Health Research Bristol Biomedical Research Unit in Nutrition, Diet and Lifestyle, Bristol, UK; Faculty of Epidemiology and Population Health, London School of Hygiene and Tropical Medicine, London, UK; School of Sport, Exercise & Health Sciences, Loughborough University, Loughborough, UK; MRC Epidemiology Unit & UKCRC Centre for Diet and Activity Research (CEDAR), University of Cambridge, Cambridge, UK; Centre for Research in Childhood Health, Exercise Epidemiology Unit, Department of Sport Sciences and Clinical Biomechanics, University of Southern Denmark, Odense, Denmark; Department of Sport Medicine, Norwegian School of Sport Sciences, Oslo, Norway; Department of Movement and Sport Sciences, University of Ghent, Ghent, Belgium; Centre for Research & Action in Public Health, Health Research Institute, University of Canberra, Canberra, ACT 2617 Australia; Federal University of Pelotas, Pelotas, RS Brazil; Department of Health and Human Physiology, University of Iowa, Iowa, USA; School of Social and Community Medicine, University of Bristol, Bristol, UK; Institute of Social and Preventive Medicine, University of Zurich, Zürich, Switzerland; Department of Exercise Science, University of South Carolina, Columbia, USA; Centre Hospitalier Universitaire Vaudois (CHUV), Lausanne, Switzerland; School of Psychological Sciences and Health, University of Strathclyde, Glasgow, Scotland; Centre for Physical Activity and Nutrition Research, Deakin University, 221 Burwood Highway, Burwood, VIC 3125 Australia; Exercise and Health Laboratory, CIPER, Faculty of Human Kinetics, University of Lisbon, Lisbon, Portugal

**Keywords:** Accelerometer, Physical activity, Sedentary, Children, Adolescents

## Abstract

**Background:**

Physical activity and sedentary behaviour in youth have been reported to vary by sex, age, weight status and country. However, supporting data are often self-reported and/or do not encompass a wide range of ages or geographical locations. This study aimed to describe objectively-measured physical activity and sedentary time patterns in youth.

**Methods:**

The International Children’s Accelerometry Database (ICAD) consists of ActiGraph accelerometer data from 20 studies in ten countries, processed using common data reduction procedures. Analyses were conducted on 27,637 participants (2.8–18.4 years) who provided at least three days of valid accelerometer data. Linear regression was used to examine associations between age, sex, weight status, country and physical activity outcomes.

**Results:**

Boys were less sedentary and more active than girls at all ages. After 5 years of age there was an average cross-sectional decrease of 4.2 % in total physical activity with each additional year of age, due mainly to lower levels of light-intensity physical activity and greater time spent sedentary. Physical activity did not differ by weight status in the youngest children, but from age seven onwards, overweight/obese participants were less active than their normal weight counterparts. Physical activity varied between samples from different countries, with a 15–20 % difference between the highest and lowest countries at age 9–10 and a 26–28 % difference at age 12–13.

**Conclusions:**

Physical activity differed between samples from different countries, but the associations between demographic characteristics and physical activity were consistently observed. Further research is needed to explore environmental and sociocultural explanations for these differences.

**Electronic supplementary material:**

The online version of this article (doi:10.1186/s12966-015-0274-5) contains supplementary material, which is available to authorized users.

## Background

Physical activity in youth is associated with many health benefits [1, 2] and physical activity behaviours established in youth are likely to be carried through into adulthood [3]. Widely accepted public health recommendations are that young people should accumulate at least 60 min of moderate to vigorous intensity physical activity (MVPA) daily [4]; however, data suggest that the majority of youth do not meet these guidelines, with approximately 80 % of 13–15 year olds worldwide insufficiently physically active [5].

Consequently, there is interest in describing how physical activity in youth varies by factors such as sex, age and weight status between samples from different countries in order to identify potential opportunities to intervene to increase physical activity. Studies using both self-report and objective measurement of physical activity have consistently shown that boys are more active than girls [6–9] and that physical activity declines and time spent sedentary increases throughout adolescence [10–12]. Physical activity is also frequently reported to be lower in overweight and/or obese children [13, 14], although the direction of causation is uncertain [15, 16]. Between-country differences in physical activity have also been described. Self-report data from the Health Behaviour in School-Aged Children study (HBSC) and the World Health Organisation (WHO) Global School-based Student Health Survey, show that the prevalence of youth aged 11–15 years meeting physical activity guidelines varies widely by country [17, 18]. In addition, two previous studies using accelerometers have shown differences in total physical activity and MVPA between Denmark, Portugal, Estonia and Norway in participants aged nine and 15 years [19] and in total physical activity, MVPA and sedentary time in children aged 11 years from Belgium, Greece, Hungary, the Netherlands and Switzerland [8], with differences in total physical activity between the highest and lowest countries generally in the range of 15–25 %.

Although objective measurement of physical activity is desirable for between-country analyses of physical activity, differences in data reduction methodology can make comparisons problematic. For example, a recent review of European studies that objectively assessed physical activity with accelerometers reported that the prevalence of children meeting guidelines for sufficiently active youth ranged between 3 and 100 % depending on the accelerometer intensity thresholds used [20]. Standardisation of accelerometer data reduction methodology is required for comparisons to be useful, and this study therefore aims to use pooled accelerometer data from the International Children’s Accelerometry Database (ICAD) to: 1) characterise variation in children and adolescents physical activity and sedentary time by age, sex and weight status; and 2) to examine to what extent the levels and patterns of children’s physical activity differ between samples from different countries located in the northern and southern hemispheres. No child and adolescent physical activity data pooling studies have incorporated samples from such a diverse range of countries using consistent measures and methodology. Exploring associations between demographic characteristics and young people’s physical activity among samples from different countries can inform the potential importance of environmental and sociocultural factors in the development of effective strategies for promoting physical activity in this age group.

## Methods

### Study design

The International Children’s Accelerometry Database (http://www.mrc-epid.cam.ac.uk/research/studies/icad) pools data on objectively measured physical activity from international studies using the Actigraph accelerometer in youth, using a standard data reduction procedure [21].

### Participants

The ICAD contains accelerometer data from twenty studies conducted in ten countries (Additional file 1: Table S1). Some of these studies include measurements taken at multiple time points for the same individual. The present analyses included baseline measurements from all 20 studies and follow-up measurements from the seven longitudinal studies and one experimental study. In addition, follow-up measurements from the control group of one of the four randomised controlled trials were also included, since for this one study it was possible to distinguish intervention and control groups. Analyses were restricted to 27,637 participants (92 % of 29,967 potential participants) who provided at least 3 days of valid accelerometer data (mean 5.3, range 3–7) from at least one time point. These 27,637 participants were aged 2.8 to 18.4 years, and between them provided 188,416 days of valid data across 35,360 time points. In analyses comparing activity levels between countries, we restricted our analyses to children 9–10 or 12–13, resulting in a sample of 10,741 participants from ten studies in eight countries.

### Measurements

#### Physical activity and sedentary time

A detailed description of the accelerometer data reduction methods used in ICAD is available elsewhere [21]. Briefly, all accelerometer data files were reintegrated to a 60 s epoch and then processed using commercially available software (KineSoft v3.3.20, Loughborough, UK) to provide physical activity outcome variables that could be directly compared across studies. Non-wear time was defined as 60 min of consecutive zeros allowing for 2 min of non-zero interruptions [7]. A valid day was defined as recording at least 500 min of measured wear time between 07:00 and 22:59 (19 % days excluded as invalid).

The primary measure of physical activity was the participants’ average accelerometer counts per minute (cpm). This was computed by calculating, for each day, the total accelerometer counts recorded divided by minutes of recording, and then averaging these daily averages across all valid days. Standard cut-points were used to define the mean daily percentage of time spent at various intensities: sedentary (≤100 cpm), light (101–2295 cpm), and moderate to vigorous (≥2296 cpm) [22]. The proportion of youth meeting physical activity guidelines is presented in two ways. Firstly, in strict agreement with current World Health Organisation physical activity guidelines [4] we present the proportion of youth who recorded at least 60 min of MVPA on every valid day measured. Secondly, we also calculated a more liberal interpretation of these guidelines, being the percentage of valid days where ≥60 min of MVPA were accumulated.

#### Anthropometry

Height and body weight were measured using standardised procedures across studies. Body mass index (BMI) was calculated as weight (kg)/height (m)^2^ and participants were categorised into normal weight, overweight and obese groups according to age and sex-specific cut points [23]. For each participant, we also computed BMI standardised by sex and by age (in 1-year age bands) in order to examine associations with a continuous measure of weight status.

#### Statistics

Linear regression was used to examine cross-sectional associations between age, sex, weight status and physical activity outcomes. All analyses were adjusted for the ICAD study from which the data was drawn and also for time of year (May-October vs. November-April, with the months inverted for the three studies from the Southern hemisphere). In order to increase statistical power we treated repeated measures in the same participants (i.e. the same child measured at different ages) as adding to the samples for cross-sectional analyses at different ages. When performing cross-sectional analyses, robust standard errors were used to adjust for the clustering of measurement waves within participants. Two-way interaction terms between age and sex were initially entered in order to characterise the physical activity of every age-sex combination relative to boys aged 5–6 years. We then fitted three-way interactions between age, sex and whether the child was normal weight versus overweight/obese.

Analyses comparing activity levels between countries were restricted to participants aged 9–15 as most measurement time points (82 %) were of participants between these ages. Country-level analyses were also restricted to ten studies from eight countries (eight studies from seven countries age 9–10, and five studies from four countries age 12–13; for both age groups, we pooled two separate studies from Melbourne, Australia). These studies were selected because they were large and/or nationally- or regionally-representative and covered a diverse range of regions from around the world. We first compared countries in terms of their absolute average activity levels, stratified by age and sex, and then compared the countries in terms of the relative within-country effect of sex, age and weight status. To make these comparisons we first standardised our activity outcome within each country in order to account for between-country differences in absolute activity levels when comparing the relative within-country effects. Separate regression models were then run in each country, and the coefficients from that regression model entered into a random-effects meta-analysis. All analyses used Stata 12.

## Results

The characteristics of the participants are summarised in Table 1. Girls were over-represented (59 % of participants), reflecting the inclusion of one large study of only adolescent girls. Excluding this study, the proportion of girls was 52 %. There was also an over-representation of data from participants aged 9–15 (82 % of measurement time points) and from those in the UK and USA (73 % of measurement time points). The prevalence of overweight/obesity (defined as an age and sex adjusted BMI equivalent to >30 kg/m^2^) was 26 % in the sample as a whole, increasing cross-sectionally across the age groups from 17 % of those aged 2–4 years to 40 % of those aged 17–18.

**Table 1:** Descriptive characteristics of study participants

		N (%) participants	N (%) measured time points
Full sample		27,637 (100 %)	35,360 (100 %)
Sex	Male	11,199 (41 %)	14,633 (41 %)
	Female	16,438 (59 %)	20,727 (59 %)
Age†	2–4 years	1044 (4 %)	1044 (3 %)
	5–6 years	2379 (9 %)	2379 (7 %)
	7–8 years	1168 (4 %)	1654 (5 %)
	9–10 years	6054 (22 %)	6910 (20 %)
	11–12 years	9981 (36 %)	11,186 (32 %)
	13–14 years	4706 (17 %)	9196 (26 %)
	15–16 years	1747 (6 %)	2342 (7 %)
	17–18 years	558 (2 %)	649 (2 %)
Weight status† ‡	Normal	20,387 (74 %)	26,222 (75 %)
	Overweight	4939 (18 %)	6238 (18 %)
	Obese	2048 (7 %)	2450 (7 %)
Country [No. studies]	Australia [*N* = 2]	2395 (9 %)	3531 (10 %)
	Belgium [*N* = 1]	257 (1 %)	257 (1 %)
	Brazil [*N* = 1]	420 (2 %)	420 (1 %)
	Denmark [*N* = 2]	1905 (7 %)	2563 (7 %)
	Estonia [*N* = 1]	643 (2 %)	643 (2 %)
	Norway [*N* = 1]	364 (1 %)	364 (1 %)
	Portugal [*N* = 1]	1070 (4 %)	1174 (3 %)
	Switzerland [*N* = 2]	742 (3 %)	742 (2 %)
	UK [*N* = 5]	10,301 (37 %)	14,424 (41 %)
	USA [N = 4]	9540 (35 %)	11,242 (32 %)

*UK* = United Kingdom, *USA* = Unites States of America

† For individuals measured more than once, the participant characteristics give age and weight status at baseline while the time point characteristics give age and weight status during the measurement period in question

‡Numbers add up to less than the total for weight status because of missing data on 1 % of participants

### Physical activity by sex, age and weight status

**Fig. 1:**
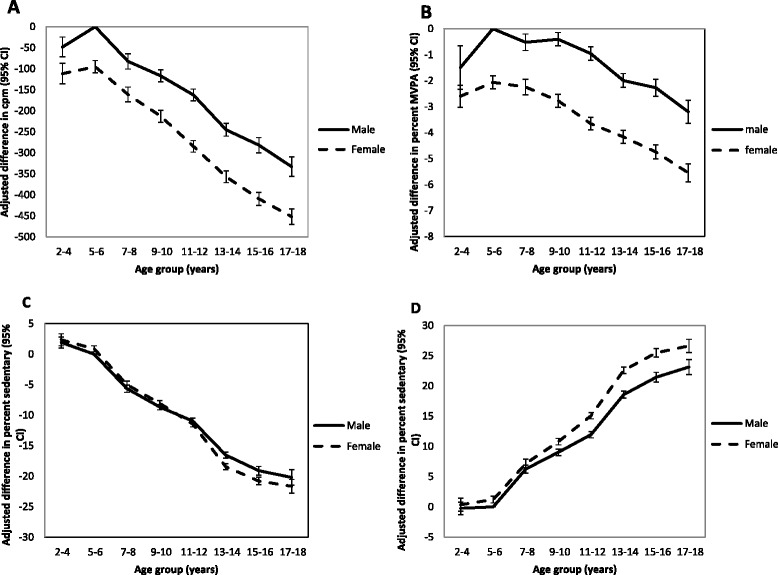
Physical activity level by age and sex, according to different activity metrics**. a** Total physical activity (accelerometer counts per minute); **b** Percentage time in Moderate to Vigorous Physical activity (MVPA); **c** Percentage time in light intensity physical activity; **d** Percentage time sedentary. CI = confidence interval, cpm = counts per minute. In all graphs, boys aged 5–6 are the reference population. All graphs present mean differences in physical activity variables, adjusting for study population and season.

Boys were more active and less sedentary than girls at all ages. Both total physical activity (Fig. 1a) and percentage of time in MVPA (Fig. 1b) were progressively lower in each age group after ages 5–6, with an average difference in physical activity of 31 cpm (29 cpm in boys, 32 cpm in girls), between each age group. Overall, this is equivalent to an average annual reduction in total physical activity of 4.2 % relative to age five (3.7 % decrease in boys, 4.6 % decrease in girls). Simultaneous with the reduction in total physical activity and percentage time in MVPA between increasing age groups, light physical activity (Fig. 1c) declined and sedentary time increased (Fig. 1d).

**Fig. 2:**
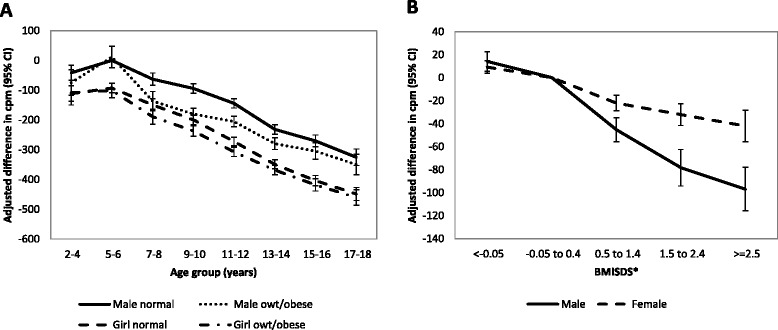
Association between weight status and physical activity. **a** Total physical activity (accelerometer counts per minute) by age, sex and weight status; **b** Dose response relationship between total physical activity and BMI by sex CI = confidence interval, owt = overweight, cpm = counts per minute

Overweight/obesity was not associated with total physical activity in the youngest age groups (age 2–6) but was consistently associated with lower total physical activity in older age groups (Fig. 2a). This association showed a dose–response relationship in both sexes, and was particularly large in boys (Fig. 2b). Similar dose–response associations and sex differences were seen for both MVPA and time spent sedentary (see supplementary material (Additional file 2: Figure S1)).

### Cross-country comparisons of physical activity

**Fig. 3:**
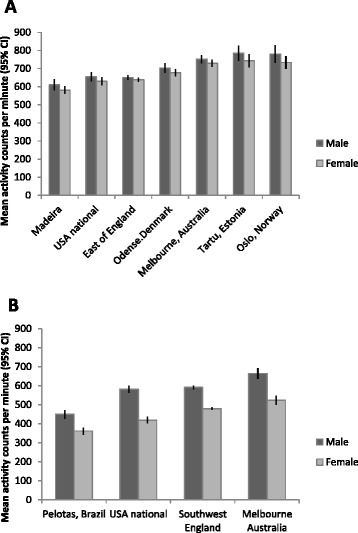
Average activity counts per minute across selected countries. **a** Level of physical activity across selected countries at age 9–10. **b** Level of physical activity across selected countries at age 12–13. CI = confidence interval. The Australian sample pools the two ICAD studies collected in Melbourne; both produced similar findings when analysed separately

**Fig. 4:**
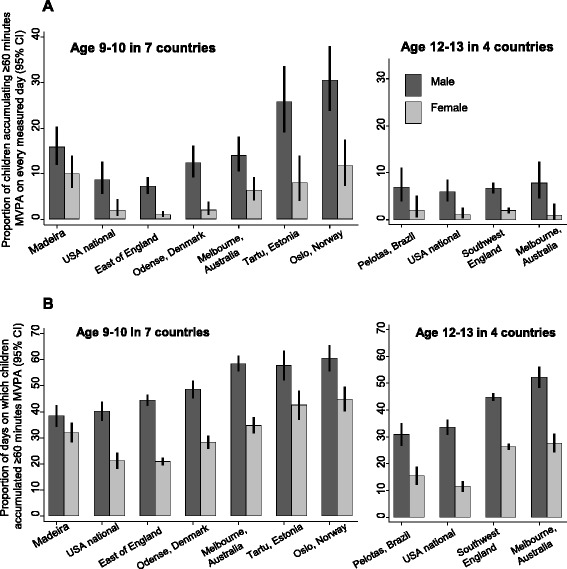
Achievement of physical activity guidelines across selected countries. **a** Average percentage of participants achieving ≥60 min of MVPA each measurement day. **b** Average percentage of days on which ≥60 min of MVPA were recorded

Average accelerometer counts per minute varied substantially between countries, both at age 9–10 (Fig. 3a) and age 12–13 (Fig. 3b). At age 9–10, the difference between the least active population (Madeira) and the most active (Oslo, Norway) was 15–20 % (156 cpm in boys (613 vs. 769) and 99 cpm in girls (561 vs. 660)). At age 12–13, even larger differences were seen (26–28 % between the highest and lowest countries). The three countries that were represented twice were ranked in the same order, with the USA least active, England intermediate and Australia most active. Alongside Australia, the three Northern European countries (Denmark, Estonia and Norway) reported the highest activity levels at age 9–10. Similar differences between countries were obtained when the results were repeated using percentage time in MVPA as the outcome (Additional file 3: Figure S2). In all countries a low percentage of participants met the guidelines of achieving 60 min of MVPA every day. Among 5–17 year olds in the ICAD database as a whole only, 9.0 % of boys and 1.9 % of girls achieved this, and across the countries presented in Fig. 3 the highest percentage recorded was 13 % among Norwegian boys (Fig. 4a). In comparison, ≥60 min of MVPA were accumulated on a substantially higher percentage of days (46 % of valid days for boys and 22 % for girls), with Norwegian boys aged 9–10 years still recording the highest values (meeting or exceeding 60 min of MVPA on 60.5 % of measured days) but with similar values recorded by boys from Estonia and Australia (Fig. 4b).

**Fig. 5:**
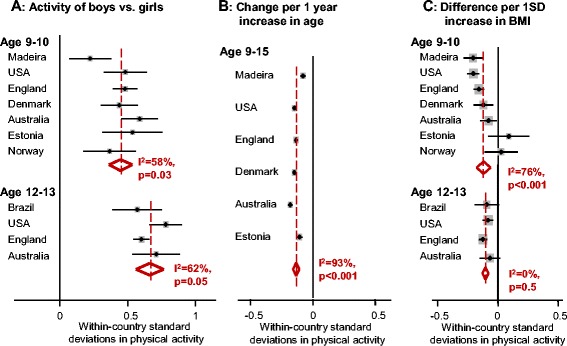
Comparison between selected countries of the relative effect of sex, age and weight status. **a** Difference in physical activity by sex in selected countries at ages 9–10 and 12–13. **b** Longitudinal change in physical activity per year of increasing age. **c** Difference in physical activity associated with higher BMI in selected countries at ages 9–10 and 12–13. SD = standard deviation. Forest plots display random-effects meta-analysis. The estimates for each country were calculated from regression models adjusted for age, sex and season, fitted to each country in turn

In all countries, boys were more active than girls. On average, activity levels among boys were 0.45 standard deviations higher than in girls at age 9–10 and 0.66 standard deviations higher at age 12–13 (Fig. 5a). Moreover, despite some evidence of between-country heterogeneity in the magnitude of this effect (I^2^-statistics = 58 % (*p* = 0.03)–62 % (*p* = 0.05)), there was no country in which this difference was not significant or where the point estimate for the difference was less than 0.2 standard deviations. Similarly although the magnitude of the cross-sectional decreases in physical activity with increasing age varied between countries, there was strong evidence of this decline in all six countries when we conducted a longitudinal analysis of the same children spanning an age range of at least 4 years (Fig. 5b). Higher BMI was associated with lower physical activity in most countries, but this difference was not significant in all samples, including the two most active samples at age 9–10 (Fig 5c).

## Discussion

This paper describes objectively measured physical activity and sedentary time in 27,637 young people from ten countries. Physical activity was consistently lower in girls than boys, was lower in overweight/obese youth, and decreased cross-sectionally each year after age five, with a corresponding increase in time spent sedentary. Substantial differences in physical activity between countries were seen for both sexes, including in the proportion meeting physical activity guidelines. Nevertheless, all countries were alike in showing the same differences in physical activity by sex, by age and (almost always) by weight status.

Sex differences in physical activity have been consistently reported in the literature and the present study confirms these findings at all ages. Previous longitudinal studies have also reported that physical activity declines through adolescence but were limited by having relatively few measurement time points and by self-reported physical activity data. A systematic review and pooled analysis [10] concluded that physical activity declined by approximately 7 % per year after the age of ten. The present study found a difference of 4.2 % per annum in overall physical activity when calculated with reference to the activity level at age five. When calculated relative to the average activity level at age 12, the annual difference was 5.8 % (4.7 % in boys, 6.8 % in girls; data not shown).

When specific physical activity intensities were examined, the lower levels of overall physical activity with increasing age were reflected by a progressive increase in the volume of sedentary time and an almost equivalent ‘displacement’ of light-intensity physical activity. An increase in accelerometer-measured sedentary time with age has been reported in other studies [24, 25], though the health consequences of this are unclear. Limited evidence supports an association between objectively measured sedentary time and metabolic health in youth in observational studies [24, 26], though prospective associations between accelerometer-measured sedentary time and the development of obesity have been reported [27]. Further studies are required to explore the consequences of displacing light-intensity physical activity with sedentary time, since engagement in even light-intensity physical activity has been shown to be beneficially associated with cardiometabolic health in adolescents [28].

The generally low levels of MVPA in this study were reflected in the small proportion of participants (9 % of boys and 2 % of girls) who met the physical activity guidelines requiring participants to accumulate ≥60 min MVPA on all measured days. In comparison, ≥60 min of MVPA were accumulated on 46 % of days for boys and 22 % for girls. This difference in outcomes highlights the difficulties in comparing studies utilising different measurement criteria. For example, a review of European studies using Actigraph accelerometers reported that 3–5 % of children met guidelines using an accelerometer threshold of >3000 cpm increasing to up to 87 % meeting guidelines using a threshold of >2000 cpm [20]. The ICAD begins to address this issue by providing accelerometer data from a substantial sample of youth reduced using a standard methodology. Adoption of this methodology by other international studies, and further extension of the ICAD, will allow a consistent picture of physical activity levels to be obtained.

Overweight/obese youth have frequently been reported to be less active than their normal weight counterparts, and again this analysis of the ICAD adds to the literature by showing that differences in physical activity by weight status are seen from 6 years of age. The direction of causation in the association between weight status and physical activity was not investigated in this analysis, but recent studies [26, 29] have suggested that lower levels of physical activity are a consequence of increased adiposity, and thus early prevention of overweight/obesity is important in maintaining physical activity levels. These studies do not, however, rule out that lower levels of physical activity may also be a contributor to increased adiposity (i.e. the relationship between physical activity and overweight is bi-directional).

Substantial differences in overall physical activity between countries were identified in both age groups investigated. There are many possible individual, social and environmental explanations for these differences that were not explored in this study. For example, active travel to school or to non-school places can be substantial contributors to overall physical activity, as are active play and independent mobility [30–33], and equally the availability of opportunities to be active or differences in the built environment may be influential on physical activity. The consistent differences by age and sex persist even though the countries differ in total physical activity levels, suggesting that these differences may to an extent be biological. However, comparison of data from Norway and the US shows that Norwegian 9–10 year old girls were, on average, as active as American boys (658 vs. 655 cpm). This suggests that the tendency of boys to be more active than girls in relative terms does not imply that the physical activity levels of girls need be ‘low’ in absolute terms, and indicates the need to identify both within- and between-population determinants of physical activity.

Strengths of this study include a substantial sample of young people across a wide age range, a consistent measurement instrument and data reduction procedures, and physical activity data from a wide variety of countries. However, although this is a substantial sample, the number of participants in some countries did not allow analysis by country, and overall the sample is skewed towards two main age groups. In addition, the countries included are not globally representative, with the US, UK and Northern Europe over-represented, few data from other geographical regions, and a dearth of data from low and middle-income countries. In addition, most samples are not nationally representative, although studies have generally sampled to be at least regionally representative. Accelerometers also have a number of limitations. When worn on the waist, as in the studies comprising the ICAD, they poorly record upper body physical activity and physical activity when cycling [34]. Thus, in countries with a high prevalence of cycling for transportation (e.g. Denmark), physical activity may be under-estimated. In addition, waist-worn accelerometers mis-classify time spent in motionless-standing as sedentary, which is not considered to be a sedentary behaviour (defined as “any waking behaviour characterised by an energy expenditure ≤1.5 metabolic equivalents (METs) while in a sitting or reclining posture” [35]), potentially leading to over estimates of the true volume of sedentary time. A further limitation is in the accelerometer thresholds used to define MVPA. Whilst the thresholds used have been shown to provide valid estimates of physical activity intensity for youth aged 5 years and above [36], more recently they have been shown to perform poorly for MVPA in children aged 4–6 years [37] and have not been validated in children under 4 years. Estimates of MVPA in these younger age groups should thus be viewed with an element of caution.

## Conclusions

The ICAD provides a large volume of accelerometer data reduced using a common protocol, which allows comparison of study samples from a number of countries. Analyses show consistent differences in physical activity by age, sex and weight status and also identify between-country differences in activity levels. Further detailed studies of the determinants of these between country differences in physical activity are required to explain the differences observed in this study. Such studies are warranted since between-country differences may show different determinants than within-population differences and give new insights into preventive mechanisms.

## Additional files

Additional file 1: Table S1. ICAD studies in alphabetic order: country of origin, design and characteristics of study participants included in the present analyses (DOC 49 kb) Additional file 2: Figure S1. Dose–response relationship between weight status, MVPA (left panel) and sedentary time (right panel) by sex. CI = confidence interval, owt = overweight, cpm = counts per minute. (PPT 138 kb) Additional file 3: Figure S2. Average percentage time in MVPA across selected countries. CI = confidence interval. The Australian sample pools the two ICAD studies collected in Melbourne; both produced similar findings when analysed separately. (PPT 210 kb) Additional file 4:
**Avon Longitudinal Study of Parents and Children (ALSPAC).** (DOC 27 kb)
