# Supplementary material for: Objectively measured physical activity and sedentary time in youth: the International children’s accelerometry database (ICAD)
Source: Int J Behav Nutr Phys Act. 2015 Sep 17;12:113. doi: 10.1186/s12966-015-0274-5 (PMC4574095; doi:10.1186/s12966-015-0274-5)
Supplement: Additional file 1: Table S1. — ICAD studies in alphabetic order: country of origin, design and characteristics of study participants included in the present analyses (DOC 49 kb) [file 12966_2015_274_MOESM1_ESM.doc]

Table S1: ICAD studies in alphabetic order: country of origin, design and characteristics of study participants included in the present analyses

| **No.** | **Full name** | **Short name** | **Country** | **Design** | **Years** | **N** | **% female** | **Age** |
| --- | --- | --- | --- | --- | --- | --- | --- | --- |
| 1 | Avon Longitudinal Study of Parents and Children | ALSPAC | UK (England) | Longitudinal (2 waves) | 2003 - 2007 | 6407 | 53% | 10 - 15 |
| 2 | Ballabeina Study | Ballabeina | Switzerland | RCT | 2008 | 322 | 48% | 3 - 6 |
| 3 | Belgium Pre-School Study | Belgium Pre-school | Belgium | Cross-sectional | 2006 - 2009 | 257 | 49% | 2 - 6 |
| 4 | Children’s Health and Activity Monitoring for Schools, UK | CHAMPS UK | UK (England) | Cross-sectional | 2006 - 2007 | 460 | 51% | 3 - 16 |
| 5 | CHAMPS: Physical Activity in Pre-school Children, US | CHAMPS US | USA | Cross-sectional | 2003 - 2006 | 428 | 51% | 2 - 5 |
| 6 | Children Living in Active Neighbourhoods | CLAN | Australia | Longitudinal (3 waves) | 2001 - 2006 | 1127 | 53% | 4 - 18 |
| 7 | Copenhagen School Child Intervention Study | CSCIS | Denmark | Natural experimental | 2001 - 2003 | 638 | 48% | 5 - 11 |
| 8 | Denmark European Youth Heart Study | Denmark EYHS | Denmark | Longitudinal (2 waves) | 1997 - 2004 | 1267 | 57% | 8 - 18 |
| 9 | Estonia European Youth Heart Study | Estonia EYHS | Estonia | Cross-sectional | 1998 - 1999 | 643 | 56% | 8 - 17 |
| 10 | Healthy Eating and Play Study | HEAPS | Australia | Longitudinal (2 waves) | 2002 - 2006 | 1268 | 53% | 4 - 15 |
| 11 | Iowa Bone Development Study | Iowa | USA | Longitudinal (4 waves) | 1998 - 2007 | 603 | 51% | 4 - 14 |
| 12 | Kinder-Sportstudie Study | KISS | Switzerland | RCT | 2005 | 420 | 52% | 6 - 12 |
| 13 | Movement and Activity Glasgow Intervention in Children | MAGIC | UK (Scotland) | RCT | 2002 | 381 | 51% | 3 - 4 |
| 14 | National Health and Nutrition Examination Survey, 2003-04, and 2005-06 | NHANES | USA | Cross-sectional | 2004 - 2006 | 4201 | 50% | 6 - 18 |
| 15 | Norway European Youth Heart Study | Norway EYHS | Norway | Cross-sectional | 1999 - 2000 | 364 | 49% | 8 - 10 |
| 16 | Personal and Environmental Associations with Children's Health | PEACH | UK (England) | Longitudinal (2 waves) | 2006 - 2009 | 1178 | 55% | 9 - 12 |
| 17 | Pelotas 1993 Birth Cohort | Pelotas | Brazil | Cross-sectional | 2006 - 2007 | 420 | 47% | 12 - 14 |
| 18 | Portugal European Youth Heart Study | Portugal EYHS | Portugal (Madeira) | Longitudinal (2 waves) | 1999 - 2008 | 1070 | 51% | 8 - 17 |
| 19 | Project Trial of Activity for Adolescent Girls | Project TAAG | USA | RCT | 2003 - 2006 | 4308 | 100% | 10 - 16 |
| 20 | Sport, Physical activity and Eating behaviour: Environmental Determinants in Young people | SPEEDY | UK (England) | Cross-sectional | 2007 | 1875 | 56% | 9 - 11 |

See Sherar *et al.* for further details about the 20 studies. RCT=randomised controlled trial; only baseline samples used in RCTs except in Project TAAG where follow-up repeat-cross-sectional samples from control schools also included.
