# Supplementary material for: Objectively measured physical activity and sedentary time in youth: the International children’s accelerometry database (ICAD)
Source: Int J Behav Nutr Phys Act. 2015 Sep 17;12:113. doi: 10.1186/s12966-015-0274-5 (PMC4574095; doi:10.1186/s12966-015-0274-5)
Supplement: Additional file 2: Figure S1. — Dose–response relationship between weight status, MVPA (left panel) and sedentary time (right panel) by sex. CI = confidence interval, owt = overweight, cpm = counts per minute. (PPT 138 kb) [file 12966_2015_274_MOESM2_ESM.ppt]

## Slide 1
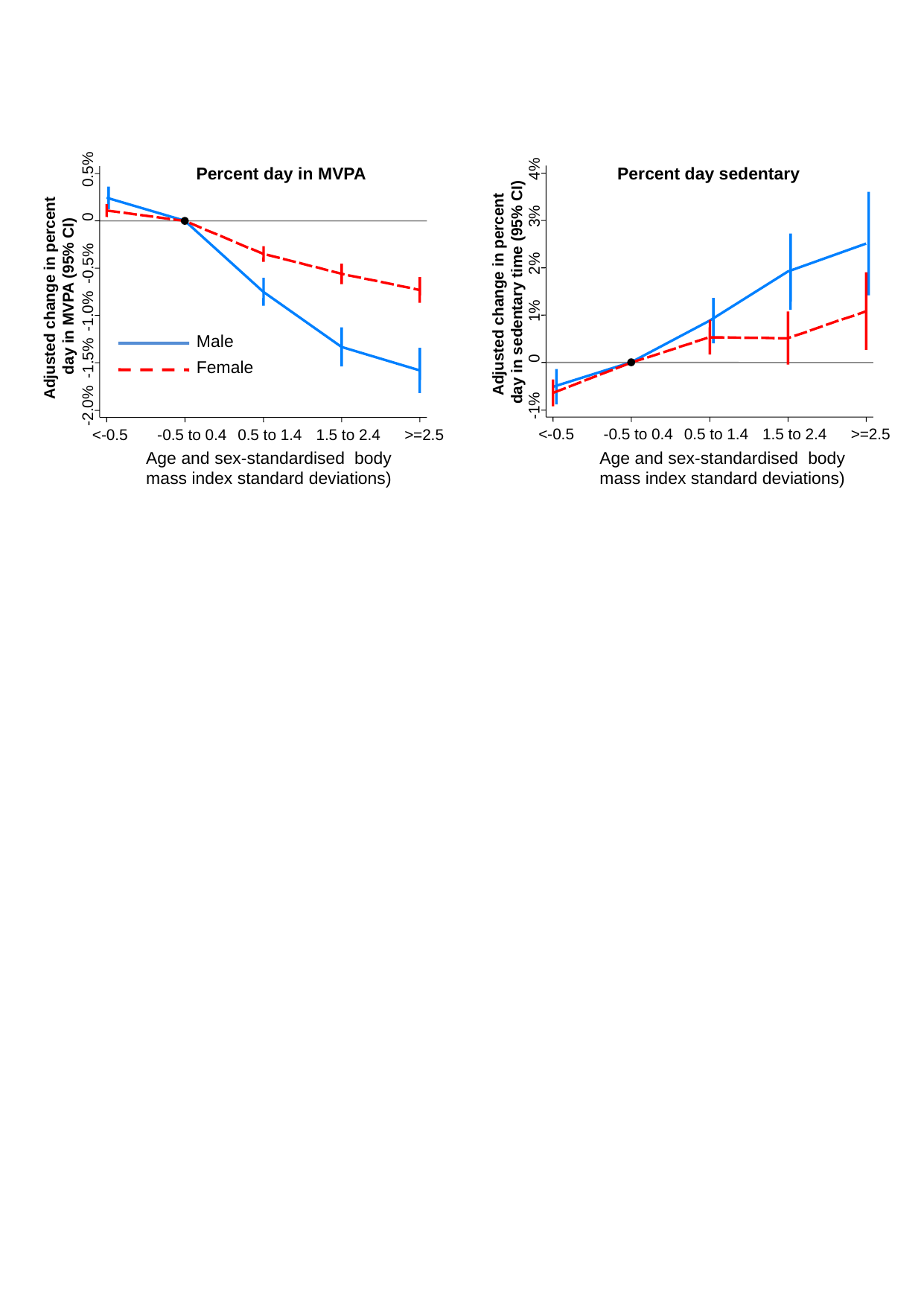

4%
0.5%
Percent day sedentary
Percent day in MVPA
3%
0
-0.5%
2%
Adjusted change in percent
 day in sedentary time (95% CI)
Adjusted change in percent
day in MVPA (95% CI)
1%
-1.0%
Male
Female
-1.5%
0
-2.0%
-1%
<-0.5
-0.5 to 0.4
0.5 to 1.4
1.5 to 2.4
>=2.5
<-0.5
-0.5 to 0.4
0.5 to 1.4
1.5 to 2.4
>=2.5
Age and sex-standardised body
mass index standard deviations)
Age and sex-standardised body
mass index standard deviations)
