# Supplementary material for: Objectively measured physical activity and sedentary time in youth: the International children’s accelerometry database (ICAD)
Source: Int J Behav Nutr Phys Act. 2015 Sep 17;12:113. doi: 10.1186/s12966-015-0274-5 (PMC4574095; doi:10.1186/s12966-015-0274-5)
Supplement: Additional file 3: Figure S2. — Average percentage time in MVPA across selected countries. CI = confidence interval. The Australian sample pools the two ICAD studies collected in Melbourne; both produced similar findings when analysed separately. (PPT 210 kb) [file 12966_2015_274_MOESM3_ESM.ppt]

## Slide 1
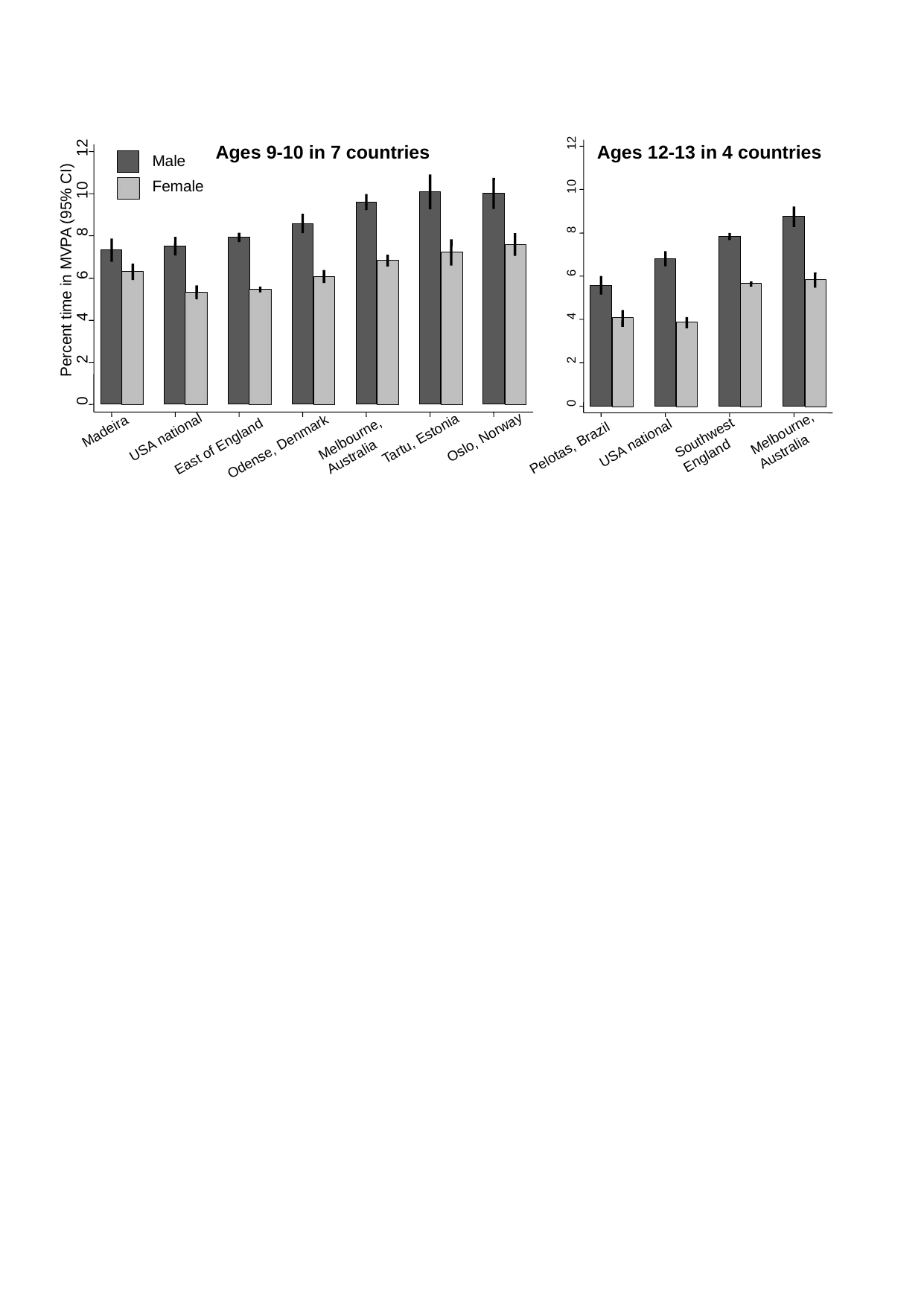

12
12
Ages 9-10 in 7 countries
Ages 12-13 in 4 countries
Male
Female
10
10
8
8
Percent time in MVPA (95% CI)
6
6
4
4
2
2
0
0
Melbourne, Australia
Madeira
Southwest
England
Melbourne,
Australia
USA national
Oslo, Norway
Tartu, Estonia
USA national
East of England
Odense, Denmark
Pelotas, Brazil
