# Supplementary material for: Objectively measured physical activity and sedentary time in youth: the International children’s accelerometry database (ICAD)
Source: Int J Behav Nutr Phys Act. 2015 Sep 17;12:113. doi: 10.1186/s12966-015-0274-5 (PMC4574095; doi:10.1186/s12966-015-0274-5)
Supplement: Additional file 4: — Avon Longitudinal Study of Parents and Children (ALSPAC). (DOC 27 kb) [file 12966_2015_274_MOESM4_ESM.doc]

**Avon Longitudinal Study of Parents and Children (ALSPAC)**

ALSPAC recruited pregnant women resident in Avon, UK with expected dates of delivery 1st April 1991 to 31st December 1992. In approximately 1998, the initial sample was expanded by recruiting eligible cases who did not join the study originally. Further details are published previously [3]. The total sample size for analyses using any data collected after the age of seven is from 15,247 pregnancies, resulting in 14,701 children alive at 1 year of age. Samples for the studies reported here were obtained from 1,567 for participants who had attended before 1100h. All the data are available through a fully searchable data dictionary at <http://www.bris.ac.uk/alspac/researchers/data-access/data-dictionary>. Ethical approval for the study was obtained from the ALSPAC Ethics and Law Committee and the Local Research Ethics Committees. ALSPAC is funded by the UK Medical Research Council and Wellcome Trust (MRC/WT Grant ref: 102215/2/13/2).
